# Participant Adherence and Contact Behavior in a Guided Internet Intervention for Depressive Symptoms: Exploratory Study

**DOI:** 10.2196/46860

**Published:** 2024-12-16

**Authors:** Oliver Thomas Bur, Thomas Berger

**Affiliations:** 1 Department of Clinical Psychology and Psychotherapy, University of Bern, Fabrikstrasse 8, Bern, 3012, Switzerland, 41 774346516

**Keywords:** internet intervention, depression, guidance, contact behavior, messages, adherence, online, intervention, digital health, therapy, participant

## Abstract

**Background:**

The number of studies on internet-based guided self-help has rapidly increased during the last 2 decades. Guided self-help comprises 2 components: a self-help program that patients work through and usually weekly guidance from therapists who support patients using the self-management program. Little is known about participants' behavior patterns while interacting with therapists and their use of self-help programs in relation to intervention outcomes.

**Objective:**

This exploratory study aimed to investigate whether the number of messages sent to the therapist (ie, contact behavior) is an indicator of the outcome, that is, a reduction in depressive symptoms. Furthermore, we investigated whether adherence was associated with outcome. Most importantly, we investigated whether different combinations of adherence and contact behavior were associated with outcome.

**Methods:**

Drawing on a completer sample (n=113) from a randomized full factorial trial, participants were categorized into 4 groups. The groups were based on median splits of 2 variables, that is, the number of messages sent to therapists (low: groups 1 and 2; high: groups 3 and 4) and adherence (low: groups 1 and 3; high: groups 2 and 4). The 4 groups were compared in terms of change in depressive symptoms (measured with the Patient Health Questionnaire-9) from pre- to posttreatment and pretreatment to follow-up, respectively.

**Results:**

On average, participants sent 4.5 (SD 3.7) messages to their therapist and completed 18.2 (SD 5.2) pages of the program in 6.39 (SD 5.39) hours. Overall, analyses revealed no main effect for participants’ messages (*H*_1_=0.18, *P*=.67) but a significant main effect for adherence on changes in depressive symptoms from pre- to posttreatment (*H*_1_=5.10, *P*=.02). The combined consideration of adherence and messages sent to the therapist revealed group differences from pre- to posttreatment (*H*_3_=8.26, *P*=.04). Group 3 showed a significantly smaller improvement in symptoms compared with group 4 (*Z*=–2.84, *P*=.002). Furthermore, there were group differences from pretreatment to follow-up (*H*_3_=8.90, *P*=.03). Again, group 3 showed a significantly smaller improvement in symptoms compared with group 4 (*Z*=–2.62, *P*=.004) and group 2 (*Z*=–2.47, *P*=.007). All other group comparisons did not yield significant differences.

**Conclusion:**

This exploratory study suggests that participants characterized by low adherence and frequent messaging do not improve their symptoms as much as other participants. These participants might require more personalized support beyond the scope of guided internet interventions. The paper underscores the importance of considering individual differences in contact behavior when tailoring interventions. The results should be interpreted with caution and further investigated in future studies.

## Introduction

Research on internet interventions has snowballed in the last 2 decades. Most studies investigated a guided self-help approach, in which a web-based self-help program is presented with therapist guidance, a minimal but regular therapist contact often via email [1]. Intensive research has shown that these interventions are effective in various clinical problems [2]. Furthermore, concerning depression, it has been shown that internet interventions effectively reduce depressive symptoms and that guided interventions tend to be more efficacious than unguided interventions [3-5]. Thereby, it seems that participants show a larger symptom improvement when they engage intensively with the program and demonstrate high adherence (usually measured by the number of clicks, completed modules, or time spent in the program) [3,6-10].

Apart from adherence to web-based programs, easily measurable aspects of guidance could also serve as indicators for the likelihood of participants’ symptom improvement. Indeed, there is anecdotal evidence that the number of messages written by participants may provide clues (personal communication from a study by Berger and colleagues [11]). For example, some participants had written many messages to the therapists and sought more contact. These participants tended to improve less than participants who wrote fewer messages to their therapist.

In this exploratory paper, we wanted to explore whether participants who differ in their adherence and contact behavior also differ in the extent of change in depressive symptoms. For this purpose, we used a completer sample of guided participants from a previous study [3]. We divided the sample based on median splits of adherence and the number of messages they sent to therapists. We then examined the 4 groups for their average rate of change in depressive symptoms. Our results may inspire future research to examine participants’ contact behavior toward therapists more closely.

## Methods

### Participants

For the current analyses, we used data from guided participants who filled in questionnaires at either posttreatment or follow-up (n=113) from a randomized full factorial trial [3]. The original study recruited 317 participants between February 2020 and February 2021 with mild to moderate depressive symptoms from Switzerland, Germany, and Austria through depression-related websites, radio interviews, self-help groups, Facebook groups, Google advertisements, and the website of the University of Bern (Bern, Switzerland). Interested individuals registered on our study website HERMES [12]. Inclusion criteria were (1) being at least 18 years of age, (2) indicating mild to moderate depressive symptoms on the Patient Health Questionnaire-9 (PHQ-9 score between 5 and 14), (3) providing written informed consent, (4) having access to the internet and an email account, and (5) providing an emergency contact. Exclusion criteria were (1) reporting a present or past psychotic or bipolar disorder or (2) indicating increased suicidal tendencies on the Suicidal Behavior Questionnaire-Revised (SBQ-R; score >7). Of note, participants taking medication or seeing a psychotherapist could take part in the study. The participants were not compensated for taking part in the study. Please see our previous publications for more details about the study design, randomization procedure, power considerations, the self-help program, treatment conditions, and study outcome measures [3,13].

### Ethical Considerations

The ethics committee of the canton of Bern (Kantonale Ethikkommission Bern) approved the HERMES study on January 20, 2020 (2019‐01795). The study was preregistered at ClinicalTrial.gov (NCT04318236). The participants could only take part in the study if they provided informed consent (Multimedia Appendix 1). They were informed that taking part in the study was at all times voluntary and that they could opt out of the study at any time without providing a reason. The informed consent covered both primary and secondary research questions and analyses. The participant data were anonymized and replaced with a code. Data could not be tracked back to an individual except with a list that included the names of the individuals and their respective codes. This list was securely locked and only accessible to the authors of this paper. The participants were not compensated for taking part in the study.

### Statistical Analyses

We used assessments at pretreatment, 8 weeks after pretreatment (posttreatment), and 16 weeks after pretreatment (follow-up). We focused on guided participants who completed the Patient Health Questionnaire-9 (PHQ-9) assessment posttreatment or follow-up. The PHQ-9 is a validated 9-item questionnaire to assess depressive symptoms [14]. We defined “adherence” as the extent to which participants used the self-help program. Following a suggestion by Donkin and colleagues [6], we calculated a composite score by averaging the *z* scores of the following indicators: number of clicks, number of topics worked on, number of completed exercises, and time spent on the program. We calculated adherence for the time from baseline to posttreatment. “Participants’ messages” were defined as the number of all messages that guided participants sent to their therapist within the self-help program from pre- to posttreatment.

For the analyses, we divided the completer sample into 4 groups based on median splits of 2 variables: adherence and participants’ messages. Therefore, the participants were categorized as showing low versus high adherence and sending few versus many messages to the therapist. We used chi-square (*χ*^2^) tests to evaluate group differences in participant characteristics at baseline for categorical data and Kruskal-Wallis *χ*^2^ tests for nonnormally distributed continuous data. Furthermore, we used Kruskal-Wallis *χ*^2^ tests to calculate the main effects of the 2 variables: adherence and participants’ messages. To compare the 4 groups concerning the PHQ-9 score changes from pre- to posttreatment and pretreatment to follow-up, we used Kruskal-Wallis tests and post hoc Dunn tests for pairwise comparisons.

## Results

### Baseline Evaluation

Of the original sample, 113 of 150 (75.3%) guided participants provided PHQ-9 data posttreatment or follow-up. The baseline characteristics of the participants and their respective tests for differences are displayed in Table 1. No pretreatment differences were observed between the groups in terms of primary outcome or participant characteristics (*P*s>.05), with one exception (concurrent psychotherapy: *χ*^2^_3_=11.19; *P*=.01).

**Table 1.** Baseline demographics and characteristics by groups.

Characteristics		Total (N=113)		Participants’ messages^a^: low (<4)				Participants’ messages: high (≥4)				Chi-square (*df*)*, P* value
				Group 1: low adherence^b^ (<0.41)		Group 2: high adherence (≥0.41)		Group 3: low adherence (<0.41)		Group 4: high adherence (≥0.41)		
Participants, n (%)		113 (100)		32 (28.3)		20 (17.7)		24 (21.2)		37 (32.7)		6.27 (3), *P*=.10
**Age**												4.91 (3), *P*=.18
	Mean (SD)	38.9 (13.6)		37.0 (12.2)		35.0 (12.7)		38.3 (14.4)		42.9 (14.4)		
	Range	20‐69		20‐63		22‐69		20‐64		21‐68		
PHQ-9^c^, mean (SD)		9.70 (2.6)		9.81 (3)		9.65 (2.8)		9.21 (2.2)		9.95 (2.4)		1.23 (3), *P*=.75
**Adherence, mean (SD)**												—^d^
	Composite	0.6 (0.9)		–0.1 (0.5)		0.9 (0.4)		0.1 (0.3)		1.4 (1)		
	Time in hours	6.39 (5.39)		2.67 (1.53)		7.0 (2.57)		4.1 (1.65)		10.76 (6.83)		
	Pages (0‐22)	18.2 (5.2)		14.3 (6.3)		21.2 (1.5)		16.0 (4.4)		21.3 (1.7)		
Messages sent, mean (SD)		4.5 (3.7)		1.6 (1.1)		2.2 (1.1)		6.2 (2.2)		7.1 (3.7)		—
**Gender, n (%)**												0.87 (3), *P*=.83
	Male	28 (24.8)		7 (6.2)		4 (3.5)		6 (5.3)		11 (9.7)		
	Female	85 (75.2)		25 (22.1)		16 (13.3)		18 (16.8)		26 (23)		
**Marital status, n (%)**												3.62 (9), *P*=.93
	Single	72 (63.7)		19 (16.8)		13 (11.5)		17 (15)		23 (20.4)		
	Married	30 (26.5)		9 (8)		6 (5.3)		5 (4.4)		10 (8.9)		
	Divorced or widowed	10 (8.9)		4 (3.5)		1 (0.9)		2 (1.8)		3 (2.7)		
	Other	1 (0.9)		0 (0)		0 (0)		0 (0)		1 (0.9)		
**Education, n (%)**												3.23 (9), *P*=.95
	Less than high school	5 (4.4)		1 (0.9)		0 (0)		0 (0)		1 (0.9)		
	High school diploma	16 (14.2)		3 (2.7)		3 (2.7)		3 (2.7)		7 (6.2)		
	University	71 (62.8)		22 (19.5)		13 (11.5)		17 (15)		21 (18.6)		
	Apprenticeship	21 (18.6)		6 (5.3)		4 (3.5)		4 (3.5)		8 (7.1)		
**Employment, n (%)**												14.23 (15), *P*=.51
	Full-time paid work	28 (24.8)		8 (7.1)		5 (4.4)		7 (6.2)		8 (7.1)		
	Part-time paid work	42 (37.2)		15 (13.3)		6 (5.3)		4 (3.5)		17 (15)		
	Unemployed	5 (4.4)		1 (0.9)		1 (0.9)		2 (1.8)		1 (0.9)		
	Student	27 (23.9)		6 (5.3)		7 (6.2)		7 (6.2)		7 (6.2)		
	At-home parent	3 (2.7)		1 (0.9)		0 (0)		2 (1.8)		0 (0)		
	Retired	8 (7.1)		1 (0.9)		1 (0.9)		2 (1.8)		4 (3.5)		
Current psychological treatment, n (%)		35 (31)		4 (3.5)		6 (5.3)		13 (11.5)		12 (10.6)		11.19 (3), *P*=.01
Current medication, n (%)		22 (19.5)		5 (4.4)		4 (3.5)		8 (7.1)		5 (4.4)		4.08 (3), *P*=.25

a Participants’ messages were defined as the number of all messages that guided participants sent to their therapist within the self-help program from pre- to posttreatment.

b Adherence was calculated with the number of clicks, number of topics worked on, number of completed exercises, and time spent on the program.

c PHQ-9: Patient Health Questionnaire-9.

d Not applicable.

### Group Differences in PHQ-9 Change From Pre- to Posttreatment

The Kruskal-Wallis chi-square test indicated no main effect for participants’ messages (*H*_1_=0.18, *P*=.67). However, there was a significant main effect for adherence (*H*_1_=5.10, *P*=.02). The Kruskal Wallis chi-square test revealed a significant effect of the 4 groups on PHQ-9 change from pre- to posttreatment (*H*_3_=8.26, *P*=.04). Post hoc pairwise comparisons revealed a significant difference between groups 3 and 4 (*Z*=–2.84, *P*=.002), with larger symptom improvement for participants with high adherence. The mean PHQ-9 change for each of the 4 groups is shown in Table 2 (posttreatment).

**Table 2.** Mean Patient Health Questionnaire-9 score change from pre- to posttreatment in the 4 groups.

Participants’ messages^a^	Adherence^b^	
	Mean (SD)	n
**Few (<4)**		
Group 1^c^: low (<0.41)	2.6 (4.1)	30
Group 2: high (≥0.41)	2.8 (4.1)	20
Total	2.7 (4.1)	52
**Many (≥4)**		
Group 3: low (<0.41)	1.8 (2.3)	24
Group 4: high (≥0.41)	4 (3.3)	37
Total	3.1 (3.2)	61
Low adherence (groups 1 and 3), total	2.2 (3.4)	56
High adherence (groups 2 and 4), total	3.6 (3.6)	57

a Participants’ messages were defined as the number of all messages that guided participants sent to their therapist within the self-help program from pre- to posttreatment.

b Adherence was calculated as a z-transformed composite score with the number of clicks, number of topics worked on, number of completed exercises, and time spent on the program.

c The groups were built using median splits (participant messages=4, adherence=0.41).

### Group Differences in PHQ-9 Change From Pretreatment to Follow-Up

Similar to the results from pre- to posttreatment, there was no main effect for participants’ messages from pretreatment to follow-up (*H*_1_=0.15, *P*=.69). Again, there was a significant main effect for adherence (*H*_1_=7.18, *P*=.007). The Kruskal-Wallis chi-square test revealed a significant effect of the 4 groups on PHQ-9 change from pretreatment to follow-up (*H*_3_=8.90, *P*=.03). Post hoc pairwise comparisons revealed a significant difference between the groups 3 and 2 (*Z*=–2.47, *P*=.007) and group 3 and 4 (*Z*=–2.62, *P*=.004), with larger symptom improvement for participants with high adherence. All other group comparisons did not yield significant differences. The mean PHQ-9 change for each of the 4 groups is shown in Table 3 (follow-up).

**Table 3.** Mean Patient Health Questionnaire-9 score change from pretreatment to follow-up in the 4 groups.

Participants’ messages^a^		Adherence^b^	
		Mean (SD)	n
**Few (<4)**	
Group 1^c^: low (<0.41)		2.4 (4.2)	23
Group 2: high (≥0.41)		4.2 (3.7)	17
Total		3.2 (4.1)	40
**Many (≥4)**	
Group 3: low (<0.41)		−0.2 (4.9)	18
Group 4: high (≥0.41)		3.5 (3.8)	36
Total		2.3 (4.5)	54
Low adherence (groups 1 and 3), total		1.2 (4.6)	41
High adherence (groups 2 and 4), total		3.7 (3.8)	53

a Participants’ messages were defined as the number of all messages that guided participants sent to their therapist within the self-help program from pre- to posttreatment.

b Adherence was calculated as a *z*-transformed composite score with with the number of clicks, number of topics worked on, number of completed exercises, and time spent on the program.

c The groups were built using median splits (participant messages=4, adherence=0.41).

### Differences in PHQ-9 Change for Concurrent Psychotherapy

The baseline evaluation showed that the number of participants in concurrent psychotherapy was unequally distributed across the 4 groups. Participants who wrote many messages to their guiding therapist were more likely to see a psychotherapist outside the study (group 3=52% and group 4=35%) than participants who wrote fewer messages to their guiding therapist (group 1=12.5% and group 2=26.5%). Based on this result, we further explored whether there were indicative differences in symptom changes within the four groups regarding whether participants were in concurrent psychotherapy. In most cases, the differences were negligible (range 0.06‐1.18 points on PHQ-9). However, there was one notable exception, that is, the difference in group 3 from pretreatment to follow-up. Within this group, participants in concurrent psychotherapy showed an improvement of 1.78 points on the PHQ-9, while those not in concurrent psychotherapy experienced a deterioration of 2.22 points.

## Discussion

### Principal Findings

This exploratory paper investigated whether adherence and contact behavior with the therapist are associated with the change in depressive symptoms. As noted in a previous study, high-adherent participants in our sample benefited more from the self-help program than low-adherent participants [3]. This was true for the improvement from pre- to posttreatment and from pretreatment to follow-up. For the difference in contact behavior, that is, participants who had written few or many messages, there was no overall difference.

However, looking more closely at the 4 groups, it was found that low-adherent participants writing many messages (group 3) showed a significantly smaller symptom improvement than group 4 from pre- to posttreatment. Furthermore, group 3 did not show a symptom improvement from pretreatment to follow-up and was significantly inferior compared with groups 2 and 4. These results suggest that low-adherent participants who write many messages may primarily seek contact with a therapist instead of trying to help themselves with a self-help program. It would not be surprising if some participants need more interpersonal support than is provided by guidance, be it because they do not have enough personal responsibility or resources to work on a program or because their motivation to change is too low still. For these participants, guided self-help interventions may not be a sufficient treatment. Thus, treatment providers might react to low-adherent and contact-seeking participants, for example, by providing extra support with telephone or face-to-face contact. Alternatively, they might refer them to other forms of treatment (eg, face-to-face psychotherapy). Apart from a need for contact, another reason for seeking much contact might be that these participants have a higher need for self-reflection and self-expression. If that were the case, treatment providers might use additional interventions to address that need, such as expressive writing tasks. To prevent participants not getting the contact they wish for, it would be possible to clarify more precisely what the idea of guidance is and how much contact they can expect. Some participants may decide against guided self-help and look for another treatment that suits their needs better.

The question of whether participants seek increased contact because they are not benefiting from the therapy, or conversely, whether increased contact hinders participants from benefiting remains unresolved and requires further investigation in future studies. Nevertheless, the behavior of group 3 may potentially serve as an indicator for treatment providers to refer participants to other treatments (eg, face-to-face psychotherapy).

Participants writing many messages seemed to be in concurrent psychotherapy more often than participants writing few, that is, 52% and 35% (groups 3 and 4), and 12.5% and 26.5% (groups 1 and 2), respectively. Interestingly, it was also found that in group 3, there was a striking difference in long-term symptom change depending on whether the participants saw a psychotherapist outside the study. Those with additional psychotherapy improved, whereas those without additional psychotherapy deteriorated. Similar to the result of the previous section, this result could indicate that guided self-help is less suitable for some participants because they need more contact or support than they receive through guidance. The participants without additional psychotherapy probably could not fully satisfy their interpersonal needs during the study, whereas participants with additional psychotherapy were probably able to fulfill them.

Our exploratory study suggests that low-adherent participants writing many messages could belong to a subgroup that does not benefit from a self-help program. These participants might need more support and contact, which is not satisfied by weekly guidance via email. Since this is an exploratory study, our findings and assumptions should be treated with caution and rather be used to systematically investigate the topic in future research.

### Strengths and Limitations

To the best of our knowledge, this is the first study to examine the interplay between program adherence and therapist contact regarding symptom improvement. It provides initial evidence that low-adherent participants seeking much contact may not benefit from guided self-help. Important limitations are the relatively small sample size and the exploratory nature of the study. Therefore, the results should be cautiously interpreted and primarily be used to build hypotheses for future studies. Furthermore, since it was exploratory, we did not correct for type I error in multiple comparisons in this study. Another limitation of this study is that we did not investigate the qualitative content of the messages regarding outcome. This might be an interesting subject for future research. There is some evidence that content of messages relates to outcome and module completion [15].

### Conclusions

This exploratory study suggests that low-adherent participants who write many messages during a guided self-help program may not benefit as much from the intervention. These individuals could have a higher need for interpersonal support, which is not met through minimal therapist guidance alone. Future research should investigate whether these participants would benefit more from alternative treatments, such as face-to-face psychotherapy, or additional interventions to address their specific needs. While our findings are preliminary, they may indicate the importance of considering participant behavior and adherence when tailoring treatment approaches.

## Supplementary material

10.2196/46860 Multimedia Appendix 1 Informed consent form.

## Abbreviations

PHQ-9: Patient Health Questionnaire-9
