# Supplementary material for: Participant Adherence and Contact Behavior in a Guided Internet Intervention for Depressive Symptoms: Exploratory Study
Source: JMIR Form Res. 2024 Dec 16;8:e46860. doi: 10.2196/46860 (PMC11665251; doi:10.2196/46860)
Supplement: Multimedia Appendix 1 [file formative-v8-e46860-s001.pdf]

b UNIVERSITÄT BERN

Einverständniserklärung

Schriftliche Einverständniserklärung zur Teilnahme an einem Studienprojekt
Bitte lesen Sie dieses Formular sorgfältig durch. Bitte fragen Sie nach, wenn Sie etwas nicht verstanden haben oder etwas zusätzlich wissen möchten. Für die Teilnahme ist Ihre schriftliche Einwilligung notwendig. Sie können dazu diese Einverständniserklärung ausdrucken, unterschreiben und per Post an untenstehende Adresse senden. Falls Sie die Einverständniserklärung durch uns zugesendet haben wollen, nehmen Sie bitte Kontakt mit uns auf.

**BASEC-Nummer (nach Einreichung):**	2019-01795
**Titel der Studie (wissenschaftlich und Laiensprache)**:	Multifaktorielle randomisierte Studie zur Untersuchung von adhärenzrelevanten Faktoren in einer internetbasierten Selbsthilfeintervention (HERMES) zur Steigerung der Problemlösefähigkeiten für Teilnehmer mit depressiven Symptomen<br><br>HERMES – Herausforderungen meistern<br>Eine internetbasierte Selbsthilfeintervention zur Förderung der Problemlösefähigkeiten und des Wohlbefindens
**verantwortliche Institution (Sponsor mit Adresse)**:	Universität Bern<br>Abteilung für Klinische Psychologie und Psychotherapie<br>Institut für Psychologie<br>Fabrikstrasse 8, 3012 Bern
**Ort der Durchführung**:	Universität Bern
**Leiter/ Leiterin der Studie am Studienort:**	Prof. Dr. Thomas Berger
**Teilnehmerin/Teilnehmer:**<br>Name und Vorname in Druckbuchstaben:<br>Geburtsdatum:<br>E-Mail-Adresse:<br><br>*Bitte notieren Sie die E-Mail-Adresse, mit welcher sämtliche Kommunikation mit dem Studienteam stattfinden soll (wir empfehlen eine anonyme Adresse).*	☐ weiblich männlich
**Notfallkontakt:**<br><br>Obwohl in dieser Studie keine negativen Effekte zu erwarten sind, müssen Sie für die Teilnahme einen persönlichen Kontakt angeben, den Sie im Falle einer Krise kontaktieren können.<br><br>Name, Vorname und Telefonnummer des Kontakts:	

- Ich wurde von dem unterzeichnenden Studienleiter schriftlich und mündlich über den Zweck, den Ablauf der Studie mit der HERMES Selbsthilfeintervention über mögliche Vor- und Nachteile sowie über eventuelle Risiken informiert.
- Ich nehme an dieser Studie freiwillig teil und akzeptiere den Inhalt der abgegebenen schriftlichen Information. Ich hatte genügend Zeit, meine Entscheidung zu treffen.
- Meine Fragen im Zusammenhang mit der Teilnahme an dieser Studie sind mir beantwortet worden. Ich behalte die schriftliche Information und erhalte eine Kopie meiner schriftlichen Einverständniserklärung.
- Ich bin mir bewusst, dass es andere Möglichkeiten zur Behandlung gibt und dass ich nebst der Teilnahme an dieser Studie jederzeit auch andere (weitere) Behandlungsmöglichkeiten in Anspruch nehmen kann.
- Ich bin einverstanden, dass die zuständigen Fachleute des Sponsors (Universität Bern) und der zuständigen Ethikkommission zu Prüf- und Kontrollzwecken in meine unverschlüsselten Daten Einsicht nehmen dürfen, jedoch unter strikter Einhaltung der Vertraulichkeit.
- Bei Studienergebnissen, die direkt meine Gesundheit betreffen, werde ich informiert. Wenn ich das nicht wünsche, informiere ich die Studienleitung per Email.
- Ich weiss, dass meine gesundheitsbezogenen und persönlichen Daten nur in verschlüsselter Form zu Forschungszwecken für diese Studie weitergegeben werden können.
- Ich kann jederzeit und ohne Angabe von Gründen von der Studienteilnahme zurücktreten. Meine weitere medizinische Behandlung ist nicht abhängig von der Studienteilnahme. Die bis zum Rücktritt erhobenen Daten werden für die Auswertung zur Studie verwendet.
- Ich bin mir bewusst, dass die in der Teilnehmerinformation genannten Pflichten einzuhalten sind. Im Interesse meiner Gesundheit kann mich die Studienleitung jederzeit von der Studie ausschliessen.

Ort, Datum	Unterschrift Teilnehmerin/Teilnehmer

Bestätigung des Studienleiters: Hiermit bestätige ich, dass dieser Teilnehmerin/ diesem Teilnehmer Wesen, Bedeutung und Tragweite der Studie erläutert wurden. Ich versichere, alle im Zusammenhang mit dieser Studie stehenden Verpflichtungen gemäss des geltenden Rechts zu erfüllen. Sollte ich zu irgendeinem Zeitpunkt während der Durchführung der Studie von Aspekten erfahren, welche die Bereitschaft der Teilnehmerin/ des Teilnehmers zur Teilnahme an der Studie beeinflussen könnten, werde ich sie/ ihn umgehend darüber informieren.

Ort, Datum	Der Studienleiter Prof. Dr. Thomas Berger<br><br>Unterschrift des Studienleiters
